# Management of and Revitalization Strategy for Megacities Under Major Public Health Emergencies: A Case Study of Wuhan

**DOI:** 10.3389/fpubh.2021.797775

**Published:** 2022-01-27

**Authors:** Xianguo Wu, Bin Chen, Hongyu Chen, Zongbao Feng, Yun Zhang, Yang Liu

**Affiliations:** ^1^Huazhong University of Science and Technology, School of Civil and Hydraulic Engineering, Wuhan, China; ^2^School of Civil and Environmental Engineering, Nanyang Technological University, Singapore, Singapore; ^3^Zhongnan Hospital of Wuhan University, Wuhan University, Wuhan, China

**Keywords:** COVID-19, epidemic disease, Chinese experience, urban management, response strategies

## Abstract

The outbreak of the COVID-19 pandemic in late 2019 has meant an uphill battle for city management. However, due to deficiencies in facilities and management experience, many megacities are less resilient when faced with such major public health events. Therefore, we chose Wuhan for a case study to examine five essential modules of urban management relevant to addressing the pandemic: (1) the medical and health system, (2) lifeline engineering and infrastructure, (3) community and urban management, (4) urban ecology and (5) economic development. The experience and deficiencies of each module in fighting the pandemic are analyzed, and strategies for revitalization and sustainable development in the future are proposed. The results show that in response to large-scale public health events, a comprehensive and coordinated medical system and good urban ecology can prevent the rapid spread of the epidemic. Additionally, good infrastructure and community management can maintain the operation of the city under the pandemic, and appropriate support policies are conducive to the recovery and development of the urban economy. These precedents provide insights and can serve as a reference for how to change the course of the pandemic in megacities that are still at risk, and they provide experience for responding to other pandemics.

## Introduction

On December 8, 2019, pneumonia of unknown cause appeared in Wuhan, and indications pointed to a novel coronavirus, SARS-CoV-2 (1). Facing the rampant spread of the disease and with increased awareness of the virus (2), Wuhan moved to close all transportation access into and out of the city on January 23, 2020, to prevent further spread of the virus (3). Two specialized hospitals, the Huoshen Mountain and Leishen Mountain Hospitals, were quickly built to receive and treat severe COVID-19 patients, and several public buildings were modified and furnished to serve as temporary hospitals with numerous beds for patients with mild symptoms (4, 5). The complete lockdown stopped the increase in confirmed cases, and centralized treatment led to a rapid decline in the number of infected people. After only 76 days, there were no remaining or additional confirmed cases for several consecutive weeks, and on April 8, 2020, Wuhan reopened for business and life, meaning that it had successfully contained the pandemic (6, 7). That the pandemic was curbed quickly demonstrates the resilience of Wuhan (8).

However, as a megacity of more than 10 million people, Wuhan needs to take various effective measures to address the problems that arose during the COVID-19 pandemic (9). Its experience holds great value to other megacities that are still in crisis due to the pandemic. The problems can be categorized into five key dimensions of urban issues as follows (10). (1) The first is medical treatment: Although Wuhan has a number of well-known hospitals, there are shortcomings in the public health system and facilities at the grassroots level that make it difficult to cope with the outbreak of a pandemic. (2) The second dimension is lifeline engineering and infrastructure: Municipal infrastructure sustained normal city operations during the pandemic period, but the spare capacity of lifeline engineering and infrastructure is insufficient, and the city's disaster control and mitigation capability still needs to be improved overall. (3) The third dimension is urban and community management: Inadequate community service resources and facilities made the workload of local community workers too heavy, making community management and life inconvenient during the pandemic. (4) The fourth dimension is urban ecology and the urban environment: Due to the high building density and floor area ratio of urban space, there is insufficient open public space with vegetation. (5) Finally, the fifth dimension is economic development: The pandemic-induced lockdown has had a considerable impact due to serious disruption of the supply chains of cities, and employment and business operations are facing severe challenges. In the postpandemic era, the most urgent task for megacities is to propose a set of scientific strategies for revitalization to compensate for shortcomings and to enhance urban disaster resilience (11).

## COVID-19 and Urban Governance

Many studies on the pandemic and cities have been conducted, focusing on multiple topics, and these studies provide valuable ideas regarding management and urban revitalization in the context of the COVID-19 pandemic. (1) Regarding medical treatment, the data show that the mortality rate of COVID-19 patients is related to whether treatment is timely and appropriate, which requires updating the information on beds and tiered treatment resources in a timely manner (12, 13). (2) With respect to lifeline engineering and infrastructure, the electrical supply capacity, water, materials and communication determine the efficiency of disaster relief activities, and a certain spare capacity of lifeline engineering and infrastructure is needed (14, 15). (3) Concerning urban and community management, the deployment and mobilization of community workers are fundamental for pandemic control, and community work should become the focus of urban management (16, 17). (4) In regard to urban ecology and the urban environment, the density of buildings and residents is positively correlated with the infection rate, which indicates that urban areas need more open spaces, such as parks and green spaces (18, 19). (5) Finally, regarding economic development, a good economy is an important underpinning for responding to the crisis caused by a pandemic, and government policies are needed to revive the economy after the pandemic (20, 21). In short, the COVID-19 outbreak has had a consequential impact on all aspects of urban life, and it has also pointed out how to establish more stable and reliable cities.

In this study, Wuhan, a typical megacity in China, is studied as an example to identify and analyze the problems that may arise as megacities face the COVID-19 crisis. Additionally, targeted development strategies are proposed to comprehensively improve the resilience of megacities. First, the impacts of the pandemic on all aspects of Wuhan's operations are assessed to identify the key issues in preventing the spread of the pandemic and in pandemic prevention and control. Then, the lessons learned from Wuhan's experience in eliminating the pandemic are summarized. Finally, considering the current situation of the city, potential strategies and measures to revitalize the city from the devastating pandemic are proposed. The findings can be used as a reference for similar megacities, suggesting strategies and measures to better respond when exposed to the impact of a pandemic disaster and to achieve sustainable urban development in the future.

## Problems Faced by Megacities During a Pandemic

The sudden outbreak of COVID-19 was a test for any megacity (22, 23). Taking Wuhan as an example, although it controlled the pandemic due to strong policies and national assistance, the city's medical system nearly collapsed during the early period of the outbreak due to the lack of experience in responding to a sudden outbreak such as that of COVID-19. During the pandemic lockdown, community management and people's livelihoods were compromised by many difficulties. Thus, there are still problems and inadequacies in the public health system, emergency response system, smart city development, urban ecological management and socioeconomic development of Wuhan.

### Public Health System

The public health system is flawed. Wuhan hosts several of the country's leading hospitals, but there are still shortcomings in its medical system, described as follows. (1) General hospitals are insufficient and unevenly distributed. Sixty percent of the general hospitals (including the nationally renowned Tongji, Xiehe, and Zhongnan Hospitals) are located in the core region, which accounts for only 1.6% of Wuhan's area. There are no general hospitals in three major urban areas and five newly built urban areas. Additionally, only five out of the 14 municipal districts have more than 7.5 beds per thousand residents. (2) The public health system is not sound, and there are insufficient medical resources for the treatment of infectious diseases. As a megacity with a permanent population of more than 11 million, Wuhan has two hospitals specializing in infectious diseases, with only 1,400 certified beds at the time of the pandemic. The number of beds per capita is 22% short of the national standard, making it difficult to withstand the severe impact of a major infectious disease (24). (3) The hierarchical diagnosis and treatment system has not been fully implemented, and the development of community-based primary medical facilities is lagging. In 2019, the number of visits to general hospitals reached 55.71 million, and the bed occupancy rate reached 94.2%, while the usage rate of primary medical facilities was less than half of that of general hospitals. Poor execution of graded treatment has further exacerbated the pressure on general hospitals during the pandemic and reduced efficiency in curbing the pandemic (25).

### Lifeline Engineering and Infrastructure

The resilience of lifeline engineering and infrastructure to emergencies is insufficient. During the rapid spread of the pandemic, municipal infrastructure in Wuhan sustained normal operations, but there is room for improvement to make the infrastructure and facilities resilient in a disaster. (1) Lifeline engineering needs more capacity for secure service. Power supply, municipal water supply and wastewater collection/treatment are lifeline engineering projects that are essential for emergency responsiveness. However, the power supply facilities are overloaded by up to 26%, and there are still six districts in Wuhan that are not fully covered by sewage utilities. (2) A comprehensive emergency response plan is lacking. During the pandemic, all public transportation services were suspended in Wuhan (2), with no timely provision of transit services to essential workers. Given the lack of commuting and logistics support, some medical staff had to spend hours walking home after working for many hours in a hospital, and a serious shortage of domestic and medical supplies occurred (26). (3) The capacity of hazardous waste treatment is inadequate. There is only one nonhazardous incineration center for the disposal of medical waste in Wuhan, with a capacity of 50 tons/day. However, the daily generation of medical waste peaked at 247 tons during the pandemic period (27). There is an enormous gap in the capacity for medical waste management.

### Community Management

The community management ability is insufficient. At the lowest level of urban management, community organizations play a crucial role in pandemic prevention and control (8). These local community organizations in Wuhan still need to be strengthened in terms of assistance and management capacity, as follows. (1) The divisions used for community units are irrational. There are presently some problems in the formation of organized communities, such as unclear boundaries, frequent changes in the constituent neighborhoods, and too many residents under one community unit (the average population of a community unit is 7,000–9,000 people), creating difficulties for community management. (2) There is a human resource shortage. The ratio of residents to community workers in certain communities exceeds 1,000:1, and the quality of the staff does not meet the requirements. In these circumstances, it is difficult to achieve good management. (3) The execution of smart community and smart city ideas is poor. The lack of smart management tools to respond to emergencies made daily life difficult during the pandemic period.

### Green and Open Space

Green and open space is scarce. The data show that high-density streets and communities with larger populations and high gross floor area ratios have higher levels of confirmed cases (28), which points to the problem of the lack of available open space in Wuhan. (1) There is insufficient urban green space. Wuhan is dotted with many mountains and lakes, but there are only a few parks, wetlands and other green spaces in the city, and only approximately 30% of neighborhoods have easy access to a park or other green space. (2) The level of open urban space is low. Due to the high density of buildings, there is not enough open space (29). With a high building density and low openness, the urban fabric is not conducive to the formation of a healthy natural ecology and will promote the spread of COVID-19 (30).

### Urban Economy

The urban economy suffered a major blow. During the pandemic, due to traffic blockades inside and outside the city, economic activities could not be carried out normally, or they were even stagnant for several days, bringing heavy losses to the economic development of Wuhan. These losses are described as follows. (1) The industrial supply chain was greatly affected. During the lockdown period, all economic activities in Wuhan were suspended. Closure measures cut off all kinds of “economic flows” (flows of people, logistics, capital, and information). (2) Employment and business operations were confronted with severe challenges. Global pandemic prevention and control led to an “oppressed consumption disaster”, and Wuhan's GDP showed negative growth in the first quarter of 2020, especially for the three demand categories of domestic consumption, capital investment, and imports and exports, which declined by more than 30% (31, 32).

## Key Strategies for Urban Revitalization After the Pandemic

**Figure 1:**
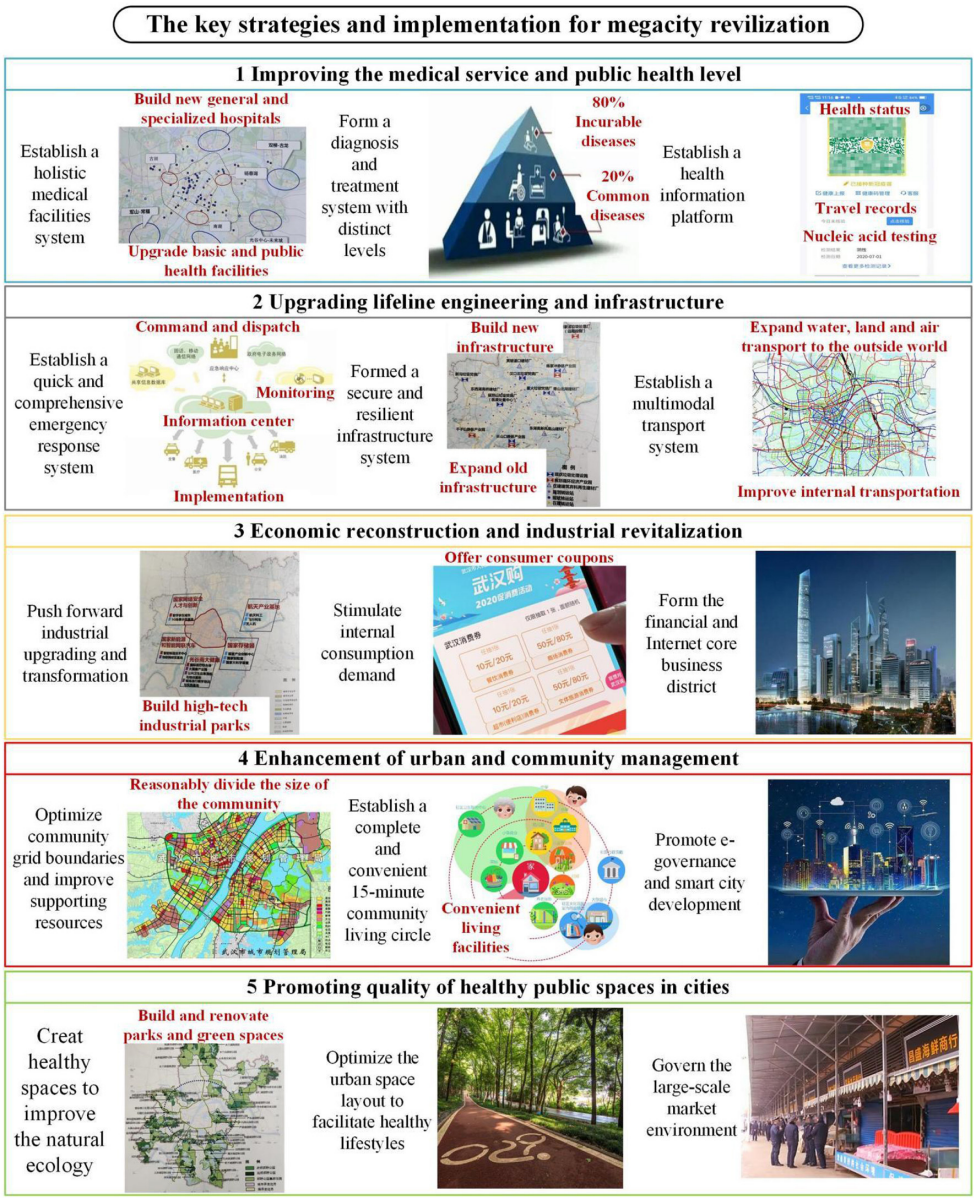
Goals of and specific measures for a megacity plan for postpandemic revitalization.

A strategy that allows a megacity to revitalize itself after a pandemic should consider the city's existing physical and management systems and aim for a comprehensively higher level of urban resilience and sustainable development (33). Taking Wuhan as an example, to compensate for the shortcomings exposed by the epidemic, this study proposes overall goals and improvement measures for the five aspects of urban development, i.e., medical treatment, urban construction, community, ecology and the economy, as shown in Figure 1.

### Improving Medical Services

In regard to improving medical services, a healthy city is a focus of public attention and a goal for sustainable development after the pandemic, and it is also the key for a megacity to restore public confidence and its image. Wuhan will focus on building its medical capacities and establishing a hierarchical diagnosis and treatment system for disease prevention and control to improve its medical treatment services and public health level.

A holistic system of medical facilities will be built to meet the needs during peacetime and wartime. Medical facilities in the city will be optimized in terms of geographical location through development by adding 20 hospitals to cover “blind spots” that lack medical services and by building two new hospitals dedicated to infectious diseases and 9 other specialized hospitals. Doing so will add as many as 20,000 beds for the treatment of infectious diseases, and the level of availability will exceed the upper limit of the national standards at 1.5 beds per 10,000 residents. The system of public health facilities will be improved by increasing the standardized medical and health facilities to 205 community facilities and building eight municipal subcenters for disease control and four first aid centers. In addition to this development program, Wuhan will have space reserved at large general hospital sites and will require both existing buildings and planned developments for public use to have associated supporting infrastructure built into them to facilitate their conversion for use in an emergency, which follows the principle of accommodating the demands of both pandemic and nonpandemic periods (34).

A coordinated, sensitive, reliable and comprehensive public health prevention system will be established. The key to curbing the spread of viruses and preventing and controlling a pandemic lies in “early detection, immediate reporting, isolation and treatment”. Wuhan will apply big data and artificial intelligence technologies to develop an “inclusive health information integration platform” for the tracking and early warning of infectious and contagious diseases. Functions such as a message service for the efficient provision of pandemic information, expert consultation and analysis, and response plans for prevention and control will be incorporated into the platform to develop a professional and modernized disease prevention and control network and to thus establish a system of prevention and control for public health events *via* dynamic monitoring and precision prevention and control.

A high-level national general medical science center will be built. Given its advantages in biomedicine and health services, Wuhan may compete for national medical research projects. Wuhan built one advanced P4 laboratory after the severe acute respiratory syndrome (SARS) epidemic and will develop a P3 laboratory for emergency services and prevention and control in the future. Teaming up with university medical schools, medical research forces will be formed to apply for state-financed medical research grants. There are also numerous biomedical and medical instrument manufacturers in Wuhan, and there is the potential to form a complete industrial chain together with scientific research institutions and hospitals in the future. Integrating these resources will sharpen Wuhan's edge across the supply chain, covering both the upper and lower streams, which will contribute to achieving its aim of becoming a national medical and health service center.

### Upgrading Lifeline Engineering and Infrastructure

Having a higher level of protection against an emergency is conducive to improving the overall capabilities of a city with regard to comprehensive disaster prevention and mitigation. For megacities, building resilience should be the focus of postpandemic development. Wuhan will establish and implement a comprehensive and effective program to increase the carrying capacity of various infrastructures and to achieve emergency responsive resilience in its lifeline engineering and infrastructure.

A secure and resilient infrastructure system will be created. All lifeline engineering will be fully accounted for and preserve redundancy to enable operational flexibility under disaster conditions. For the electricity and water supply, availability in case of an emergency should be considered. Wuhan will add four high-standard substations for power supply and three water treatment plants for emergency service to increase the power supply by 60% and to provide an additional emergency water supply of 1 million m^3^, creating high-level redundancy in the power and water supply for emergency use. Wuhan will also accelerate the construction of 12 large sewage treatment plants to increase the daily sewage treatment capacity by 1.143 m^2^ or 40% (35). Based on 100% harmless treatment of municipal solid waste, Wuhan will build a batch of new waste treatment facilities to comprehensively improve the capacity to dispose of household waste, construction waste, hazardous waste and medical waste. In addition, 16,000 5G base stations will be deployed to provide infrastructure for the development of smart cities and related industries (36).

A comprehensive emergency system will be established to respond to emergencies in a timely manner. Holistic emergency response plans for various kinds of disasters will be developed, with three stages: a pre-event stage in which preparation is made, an in-event stage in which interventions to sustain services are implemented, and a postevent stage in which disrupted operations are restored. The plans for the pre-event preparation stage will address the spatial layout and activation sequence of the facilities for disaster prevention and mitigation based on risk assessment, and they will include associated development requirements. Those for the in-event and intervention stages will define the requirements for emergency use facilities to be converted from normal operations and to wartime operations to withstand the disaster and offer services. The plans for the postevent and recovery stages will clarify the schedule of restoration and associated requirements regarding the replacement and repair of various emergency facilities. In addition, plans to restore various systems for domestic life, business operations, transportation, and other services will be prepared.

A multimodal transport system will be established. Wuhan will proactively push regional transport infrastructure development, including six high-speed rail lines, one freight airport and 10 waterway facilities for shipping along the Yangtze River, to consolidate its position as a transportation hub in Central China. In addition, 600 kilometers of intercity expressways will be built to connect Wuhan with surrounding cities and towns, and nine new logistics centers will be built. Wuhan will have a metropolitan high-speed logistics network and will seek to become a center of logistics in Central China. Wuhan will also extend its urban rail transit network by another 400 kilometers and build nine river-crossing passages and 45 km of urban arterial roads. It will comb through and regulate the logistics system and shared transportation sector and address delivery and “last mile” problems. By further improving both local and regional transport facilities and services, a national-level multimodal transportation hub will be developed.

### Enhancing Urban and Community Management

Grid-oriented community management methods have played an important role in fighting the pandemic (37), and the development of a sound and intelligent community service and urban management system is a key aspect of megacity governance for the postpandemic era. Wuhan will normalize and standardize community development and management and develop a smart community and city management platform to improve the level of community and city management.

Supporting resources will be improved, and community grid boundaries will be optimized. The size of communities will be adjusted and optimized, and the population will be maintained at approximately 3,500–5,000. Considering road separation and functional integrity, the boundaries of communities should be reasonably demarcated. The assignment of management staff and the provision of facilities will be improved. In addition to population registration, functions such as urban management and economic surveys will be incorporated into a community grid-based integrated information platform, one of the key projects to be implemented, for the comprehensive management of information on residents, land area, properties and industries to realize fully functional community grid management over the entire jurisdiction.

A complete community will be developed to improve living circles within a 15-min walking radius. A reasonable comprehensive community development index system will be established as the basis and standard for the construction of new and old communities. Based on the norm that for every 20,000 square meter floor area of housing, senior homes and services, sports facilities, medical services, cultural facilities, grocery stores and community management operations are essential and required, living circles within a 15-min walking radius will be developed. On this basis, a program for improvement and rehabilitation projects in 340 ordinary urban communities and at 114 historic street sites will be carried out.

The development of smart communities and smart cities will be further promoted (38). A public information service platform supporting data unification and sharing with access to multiple institutional levels of government authorities, street offices and community organizations will be developed to support social services, urban operations and government administration. Artificial intelligence will be used to refine and standardize the data acquired by front-end smart detectors to generate big data that can be used for efficient correlation analysis as well as granulated data for different application scenarios to digitize and virtualize all urban elements and thus provide real-time and all-status visualization for synergy and smart operation management.

### Promoting the Quality of Healthy Public Spaces in Cities

Spatial representations of the spread of the virus during the pandemic show that open and vegetated public spaces can effectively slow and flatten the curve of confirmed cases. Megacities should make more effort to improve the ecology and natural environment in urban areas after the pandemic to create high-quality and healthy public spaces in cities (39, 40). Wuhan will build new parks and rehabilitate old parks, and it will create green spaces with associated facilities to establish more high-quality, open and green urban spaces, contributing to a culture of healthy life habits and improving the urban ecology and urban environment.

Green and healthy spaces will be created to improve the natural ecology in the city. Relying on the city's unique landscape resources, efforts will be made to continue the development of 14.2 km^2^ of river-view parks along the Yangtze River and 20 km of ecological corridors in the hilly areas of the municipality, and a large theme park will be built to enhance the attractiveness of the city to tourists. Ninety countryside parks will be built where local conditions are appropriate to provide healthy leisure places and a better urban ecology and urban environment. In addition, the development of mini-parks and greenways in the city will be accelerated for higher coverage by a network of green spaces in the central urban areas. Specifically, 954 parks and 530 km of greenways are being planned and will provide a full range of healthy and diversified natural spaces to people in Wuhan. As a result, the negative effects of high-density buildings can be alleviated, and reserves for emergency evacuation and disaster prevention can also be created.

Large markets will be comprehensively overhauled to remove potential public health hazards. In accordance with the principle of distributing markets in three towns in a balanced manner and equipping them with complete utilities and service functions, Wuhan will work proactively to develop a layout system for large markets in the city. Existing large markets for groceries and food products and various specialized markets in the main urban districts and other densely populated areas will be systematically checked to identify those violating hygiene and health codes to promote risk reduction in grocery/food markets and specialized markets. Efforts will also be made to relocate and rehabilitate markets with hidden hygiene and/or safety hazards to achieve an overall improvement in the context surrounding existing large markets with respect to urban functions, transportation, facilities and the environment.

### Economic Reconstruction and Industrial Revitalization

Economic reconstruction is the main driver for improving overall urban strength, and it lays the foundation for a megacity to restore its competitiveness and attractiveness. In full alignment with the postpandemic policy direction, Wuhan will vigorously promote high-end and innovative industries to form a high-quality, sustainable economic structure that contributes to the national center city development strategy (41).

Industrial upgrading and transformation will be pushed forward to achieve long-term sustainable economic development. In the postpandemic period, Wuhan will make a greater effort to develop an extensive health system of industries integrating medical care, testing, rehabilitation, and laboratory services. Additionally, it will cultivate an emergency industrial park that integrates emergency-related functions such as technology research and development, emergency product manufacturing, warehouse services for materials, and exercises and training for emergency response, and it will develop a smart logistics industry linking the industries that offer unmanned warehousing, unmanned sorting, logistics robots and drone delivery. Wuhan will also accelerate the development of five national bases for the chip manufacturing, cloud service, new energy vehicle, aerospace, and medicine and health sectors. It will drive the economy with new types of investment and consumption, with a focus on overall economic sustainability and development in the intermediate term and the long term.

The development of new infrastructure will be promoted, and consumer demand will be stimulated across the board. Wuhan plans to create a digital twin by promoting investment in “new infrastructure” and the development of related industries and supply chains, fostering 5G industries and the industrial Internet of Things. In addition, assistance programs will be implemented to support emerging businesses and boost employment and consumption.

The status of urban economic centers will be improved, and high-tech innovation resources will be introduced. Other tasks to be completed include comprehensively expanding the economic influence of the Wuhan metropolitan area, attracting financial institutions and large enterprises to set up headquarters in Wuhan, and establishing a comprehensive national industrial innovation center as well as national laboratories for medicine, new energy and other purposes.

## Discussion

The COVID-19 outbreak represents an extreme challenge to the public health and disease prevention and control system of any megacity. Considering the evolution of the global pandemic, many megacities still fall far short of achieving the goals of being healthy, smart and sustainable with respect to public health, emergency response facilities and systems, economic industries, municipal infrastructure management and ecology and the environment. Taking Wuhan as an example, this study reviews the weaknesses of this megacity in dealing with the pandemic to propose sustainable development strategies and measures for implementation by any megacity in the postpandemic period, with insights regarding the necessary actions for a megacity to build its resilience.

The strategies and technical routes proposed in this paper might be far from sufficient to strengthen the global fight against the pandemic, but they are nevertheless significant for changing the tragic trajectory of the pandemic in megacities. Given that curbing COVID-19 is likely to be a protracted battle, it is important for managers to take certain steps to improve the hygiene and health conditions of their cities to make them more flexible and effective in addressing COVID-19 or other pandemics in an unpredictable future.

## Data Availability Statement

The original contributions presented in the study are included in the article/supplementary material, further inquiries can be directed to the corresponding author/s.

## Author Contributions

XW, BC, and YL contributed to conception and design of the study. HC organized the database. ZF performed the statistical analysis. BC wrote the first draft of the manuscript. XW, HC, ZF, YZ, and YL wrote sections of the manuscript. All authors contributed to manuscript revision, read, and approved the submitted version.

## Funding

This work was supported by the National Natural Science Foundation of China (Grant Nos. 51378235, 71571078, and 71871171), National Key R&D Program of China [Grant No. 2016YFC0800208], Science and Technology Planning Project of the Hubei Province in 2020 (Grant No. 202041), and Zhongnan Hospital of Wuhan University Science, Technology and Innovation Seed Fund, Project CXPY2020013.

## Conflict of Interest

The authors declare that the research was conducted in the absence of any commercial or financial relationships that could be construed as a potential conflict of interest.

## Publisher's Note

All claims expressed in this article are solely those of the authors and do not necessarily represent those of their affiliated organizations, or those of the publisher, the editors and the reviewers. Any product that may be evaluated in this article, or claim that may be made by its manufacturer, is not guaranteed or endorsed by the publisher.
